# Rapid identification and quantification of *Campylobacter coli *and *Campylobacter jejuni *by real-time PCR in pure cultures and in complex samples

**DOI:** 10.1186/1471-2180-11-113

**Published:** 2011-05-22

**Authors:** Mily Leblanc-Maridor, François Beaudeau, Henri Seegers, Martine Denis, Catherine Belloc

**Affiliations:** 1 LUNAM Université, Oniris, UMR 1300 Biologie, épidémiologie et analyse des risques, Nantes, F-44307, France; 2 INRA, Nantes, F-44307, France; 3 Anses, French Agency for Food, Environmental and occupational Health and Safety, Unité Hygiène et Qualité des Produits Avicoles et Porcins, BP 53, Ploufragan, F-22440, France

## Abstract

**Background:**

*Campylobacter *spp., especially *Campylobacter jejuni *(*C. jejuni*) and *Campylobacter coli *(*C. coli*), are recognized as the leading human foodborne pathogens in developed countries. Livestock animals carrying *Campylobacter *pose an important risk for human contamination. Pigs are known to be frequently colonized with *Campylobacter*, especially *C. coli*, and to excrete high numbers of this pathogen in their faeces. Molecular tools, notably real-time PCR, provide an effective, rapid, and sensitive alternative to culture-based methods for the detection of *C. coli *and *C. jejuni *in various substrates. In order to serve as a diagnostic tool supporting *Campylobacter *epidemiology, we developed a quantitative real-time PCR method for species-specific detection and quantification of *C. coli *and *C. jejuni *directly in faecal, feed, and environmental samples.

**Results:**

With a sensitivity of 10 genome copies and a linear range of seven to eight orders of magnitude, the *C. coli *and *C. jejuni *real-time PCR assays allowed a precise quantification of purified DNA from *C. coli *and *C. jejuni*. The assays were highly specific and showed a 6-log-linear dynamic range of quantification with a quantitative detection limit of approximately 2.5 × 10^2 ^CFU/g of faeces, 1.3 × 10^2 ^CFU/g of feed, and 1.0 × 10^3 ^CFU/m^2 ^for the environmental samples. Compared to the results obtained by culture, both *C. coli *and *C. jejuni *real-time PCR assays exhibited a specificity of 96.2% with a kappa of 0.94 and 0.89 respectively. For faecal samples of experimentally infected pigs, the coefficients of correlation between the *C. coli *or *C. jejuni *real-time PCR assay and culture enumeration were R^2 ^= 0.90 and R^2 ^= 0.93 respectively.

**Conclusion:**

The *C. coli *and *C. jejuni *real-time quantitative PCR assays developed in this study provide a method capable of directly detecting and quantifying *C. coli *and *C. jejuni *in faeces, feed, and environmental samples. These assays represent a new diagnostic tool for studying the epidemiology of *Campylobacter *by, for instance, investigating the carriage and excretion of *C. coli *and *C. jejuni *by pigs from conventional herds.

## Background

*Campylobacter *spp. are recognized as the leading human foodborne pathogens in developed countries [1,2]. Within the genus *Campylobacter*, the thermophilic species *Campylobacter jejuni *(*C. jejuni*) and *Campylobacter coli *(*C. coli*) are the most frequently associated with illness, accounting for over 95% of infections (respectively responsible for 80 to 85% and 10 to 15%) [2]. These two species commonly live in the intestinal tract of birds and mammals, including food production animals and pets, without causing clinical signs [3]. Livestock animals carrying *Campylobacter *pose an important risk for human infection from contamination of carcasses at slaughter, of milk, and water contaminated by livestock wastes and slurries [4-6]. Research carried out in Europe has shown the dominance of *C. jejuni *in animal intestinal tracts, for example, broiler chickens, cattle, and wild-living mammals and birds [2,7,8]. Pigs are known to be frequently infected with *Campylobacter *(prevalence between 50% and 100%), to exhibit high counts of this pathogen in their faeces (ranging from 10^2 ^to 10^7 ^Colony Forming Units (CFU) of *Campylobacter *per gram), and to show a dominance of *C. coli *[9-11]. Nevertheless, some studies have found a dominance of *C. jejuni *in pigs and of *C. coli *in chickens [12-15]. Given these contradictory data, the risk of foodborne disease associated with animal species is not clear. In terms of risk assessment, the ability to differentiate and quantify these two species is essential to describe more precisely the presence of *Campylobacter *in livestock animals.

The identification of *Campylobacter *using conventional methods is slow (culture-based methods can take up to five days) and problematic due to their fastidious growth requirements and biochemical inertness [16,17]. Moreover, the detection of *C. coli *and/or *C. jejuni *in complex substrates like faeces or environmental samples is difficult as the culture conditions have to be selective enough to avoid overgrowth from competiting organisms. Additionally these bacteria may enter into a viable but nonculturable state (VBNC) [18]. The correct differentiation of thermophilic *Campylobacter *spp., especially *C. coli *and *C. jejuni*, by phenotypic tests is difficult and hippurate hydrolysis test used to distinguish these two species is often problematic [19]. Furthermore, *C. jejuni *may also coexist with *C. coli *in pigs, but at 10-100-fold lower numbers than *C. coli *[10,11,20], so *C. jejuni *will be less frequently isolated from such samples because only a few colonies are identified to the species level with conventional culturing and biochemical testing techniques. Molecular methods are an alternative to the bacteriological method for the detection of *C. coli *and *C. jejuni *in various substrates [1,17,21-24]. Real-time PCR has provided a reliable tool to detect and to quantify *C. jejuni *and/or *C. coli *in pure culture [25], in poultry, milk, or water [26,27], and in complex substrates like food products [28-30] and faecal samples [20,31-33]. However, of the real-time PCR techniques developed, none were capable of differentiating and quantifying *C. coli *and *C. jejuni *directly from pig faecal, feed, and environmental samples.

The present study aimed to develop a species-specific real-time PCR method to detect and quantify *C. coli *and *C. jejuni *directly in pig faecal, feed, and environmental samples. The first step in the development of the assay was the definition of the multiplex PCR assay to quantify *C. coli *and *C. jejuni *isolates from bacterial cultures. These real-time PCR assays were coupled with a modified DNA extraction protocol and then examined for their ability: (i) to evaluate DNA purification with different parameters (potential presence of PCR inhibitory compounds, DNA yield, and reproducibility), (ii) to measure the sensitivity and the specificity of PCR-based detection in faecal samples, and (iii) to detect *C. jejuni *among predominant *C. coli*. Finally, the last step was the application of the real-time PCR assays to detect and quantify *C. coli *and *C. jejuni *in complex substrates like feed, environmental samples, and faeces from experimentally as well as naturally infected pigs. The bacterial culture was used as a gold standard for their validation.

## Results

### Specificity, sensitivity and linear range of the real-time PCR assays

The specificity of each primers-probe set for the detection of *C. coli *and *C. jejuni *was tested against different strains of *C. coli *(n = 77) and *C. jejuni *(n = 54), all of which were correctly identified. Moreover, no signal was observed for any of the other *Campylobacter *species tested as well as for a range of bacteria, which could be present in faecal samples or responsible for diarrhoea in pigs and humans (Table 1). Finally, the specificity of each real-time PCR assay was characterized for samples using the stool-screening strategy described previously by Lagier *et al. *(2004) [33]. The DNA extracted from the 30 *Campylobacter-*negative faecal, feed, and environmental samples and examined in duplicate with each real-time PCR assays produced threshold cycle (Ct) values ≥ 42 when 5 μL of extracted DNA was used as the starting template. All samples in which both duplicates had a Ct value below this threshold were regarded as positive.

**Table 1:** List of strains used for the validation of specificity of *Campylobacter coli *and *Campylobacter jejuni *real-time PCR assays

Bacterial species (*n*)	Name or origin of strain	*C. coli *real-time PCR identification	*C. jejuni *real-time PCR identification
*Campylobacter coli (2)*	CCUG 11283, CIP 7081	Positive	Negative
*C. coli *pig isolates (*25*)	Anses, ENVN-INRA	Positive	Negative
*C. coli *poultry isolates (*25*)	Anses, ENVN-INRA	Positive	Negative
*C. coli *human isolates (*25*)	Anses, CNR-CH	Positive	Negative
*Campylobacter jejuni *subsp *jejuni *(*3*)	CCUG 11284, NCTC 11168, NCTC 81176	Negative	Positive
*C. jejuni*	CIP 103726	Negative	Positive
*C. jejuni *poultry isolates (*25*)	Anses, ENVN-INRA	Negative	Positive
*C. jejuni *human isolates (*25*)	Anses, ENVN-INRA, CNR-CH	Negative	Positive
*Campylobacter fetus *subsp *fetus *(*2*)	CCUG 68231, CIP 2595396	Negative	Negative
*Campylobacter fetus *subsp *venerealis*	CCUG 33899	Negative	Negative
*Campylobacter hyointestinalis*	CCUG 14169	Negative	Negative
*Campylobacter lari *(*3*)	CCUG 23947, ATCC 35222, CIP 107080	Negative	Negative
*Campylobacter upsaliensis *(*2*)	CCUG 14913, CIP 400	Negative	Negative
*Campylobacter sputorum*	CIP 103749	Negative	Negative
*Helicobacter canis*	CIP 104753	Negative	Negative
*Helicobacter felis*	CIP 104382	Negative	Negative
*Helicobacter mustelae*	CIP 103759	Negative	Negative
*Helicobacter pullorum*	CIP 104787	Negative	Negative
*Helicobacter pylori *(*3*)	CIP 103995, CIP 26695, CIP 101260	Negative	Negative
*Wolinella succinogenes*	CCUG 13145	Negative	Negative
*Arcobacter butzleri*	CCUG 30485	Negative	Negative
*Arcobacter cryaerophilus*	CIP 104014	Negative	Negative
*Listeria monocytogenes *(*3*)	CIP 103575, ATCC 895807, ATCC 19115	Negative	Negative
*Listeria innocua *(*3*)	CCUG 15531, ENVN-INRA	Negative	Negative
*Salmonella enterica *serovar *enteridis*	ENVN-INRA	Negative	Negative
*S. enterica *serovar *typhimurium*	ATCC 13311	Negative	Negative
*Enterococcus faecalis *(*2*)	CIP 103013, CCUG 19916	Negative	Negative
*Escherichia coli*	V517	Negative	Negative
*Pseudomonas aeruginosa *(*2*)	ENVN-INRA	Negative	Negative
*Enterobacter aerogenes *(*2*)	ENVN-INRA	Negative	Negative
*Staphylococcus aureus *(*2*)	ENVN-INRA	Negative	Negative
*Yersinia ruckeri*	ATCC 29473	Negative	Negative
*Y. ruckeri *fish isolates (*5*)	ENVN-INRA	Negative	Negative

*n*, number of strains

NCTC, National Collection of Type Cultures (Colindale, UK); CCUG, Culture Collection University of Göteborg (Göteborg, Sweden); ATCC, American Type Culture Collection (Manassas, Va); CIP, Collection of the Pasteur Institute (Paris, France); Anses: Strains from the collection of the French Agency for Food Safety (Ploufragan, France); CNR-CH: Strains isolated from the collection of the French National Reference Center for *Campylobacter *and Helicobacter (Bordeaux, France); ENVN-INRA: Strains isolated from our in-house collection

**Figure 1:**
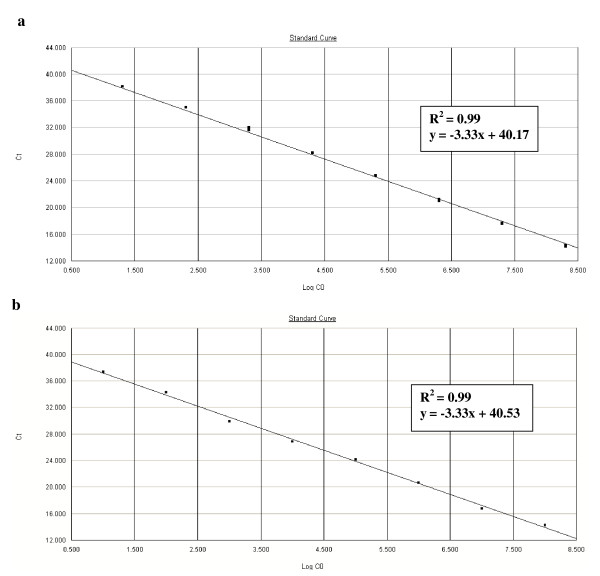
**Dynamic range and sensitivity of the *Campylobacter coli *and *Campylobacter jejuni *real-time PCR assays**. Standard curves of 10-fold serial dilution of standard DNA of (**a**) *C. coli *CIP 70.81 (from 0.3 × 10^1 ^to 3.0 × 10^8 ^genome copies per PCR reaction) and of (**b**) *C. jejuni *NCTC 11168 (from 10^1 ^to 10^8 ^genome copies per PCR reaction) are reported, each dot representing the result of duplicate amplification of each dilution. The coefficients of determination R^2^ and the slopes of each regression curve are indicated. The standard curves are obtained by correlation of the threshold cycle values (Ct) and log_10 _input genome copy number (Log CO) from the amplification plot.

**Figure 2:**
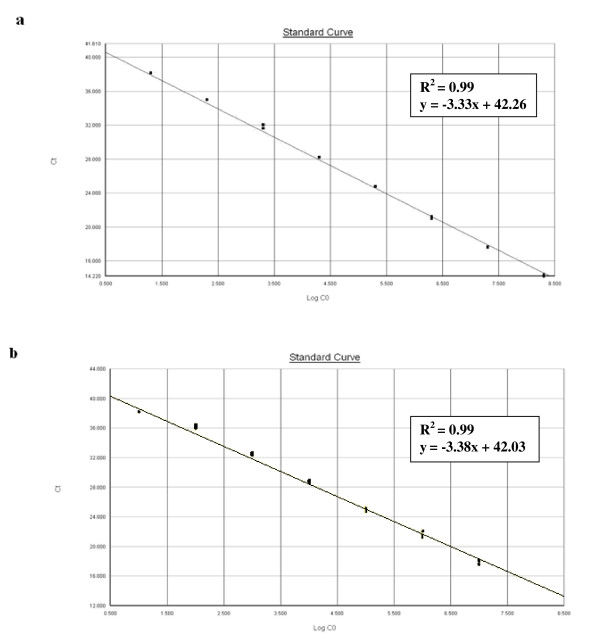
**Dynamic range and sensitivity of the *Campylobacter coli *and *Campylobacter jejuni *real-time PCR assays with samples containing roughly equal genome copies of both species**. The linear range of each real-time PCR assay was determined using *C. coli *CIP 70.81 and *C. jejuni *NCTC 11168 standard DNA together. Standard curves of 10-fold serial dilution of both *C. coli *and *C. jejuni *standard DNA (roughly from 10^1 ^to 10^8 ^genome copies of each species per PCR reaction) by (**a**) *C. coli *real-time PCR assay and by (**b**) *C. jejuni *real-time PCR assay are reported, each dot representing the result of duplicate amplification of each dilution. The coefficients of determination and the slopes of each regression curve are indicated. The standard curves are obtained by correlation of the threshold cycle values (Ct) and log_10 _input genome copy number (Log CO) from the amplification plot.

To determine the linear range of the real-time PCR assay, standard curves of the template DNA, in units of genome copy number, were generated for *C. coli *(Figure 1a) and for *C. jejuni *(Figure 1b). We observed a strong linear correlation (R^2 ^values were all equal to 0.99), providing an accurate measurement over a large variety of starting target amounts (Figure 1). The detection limits of the real-time PCR assays for genomic DNA were three genome copies per PCR reaction for *C. coli *and ten genome copies per PCR reaction for *C. jejuni *(Figure 1). Moreover, the reaction is reliable with a detection limit of ten genome copies for the samples containing both *C. jejuni *and *C. coli *DNA (Figure 2) and for 10 successive real-time PCR assays. The standard curves showed linearity over the entire quantitation range and spanned eight and seven orders of magnitude for *C. coli *and *C. jejuni *detection, respectively. Finally, the real-time PCR assays had an efficiency of 99% to detect *C. jejuni *and *C. coli *whether alone (Figure 1) or together in a same sample (Figure 2).

### Precision of the C. jejuni and C. coli real-time PCR assays

To obtain values for the intra- and inter-assay variation of each real-time PCR assay, purified genomic DNA from 10^1 ^to 10^8 ^genome copies per PCR reaction was subjected to each real-time PCR in ten duplicates, with 10 different mixes performed on different runs. The results are presented in Table 2. The coefficients of variation (CV) of the Ct values for the ten different intra-assay experiments ranged from 0.81 to 2.27% for *C. coli *real-time PCR and from 0.35 to 5.63% for *C. jejuni *real-time PCR. The mean standard curves were *y *= -3.33*x *+ 40.17 with R^2 ^= 0.99 for *C. coli *PCR and *y *= -3.33*x *+ 40.53 with R^2 ^= 0.99 for *C. jejuni *PCR. The CV of the Ct values for the inter-assay variation ranged from 1.52 to 4.89% and from 0.67 to 2.65%, respectively for *C. coli *and *C. jejuni *real-time PCR assays. The mean standard curves were *y *= -3.39*x *+ 42.70 for the *C. coli *real-time PCR and *y *= -3.20*x *+ 40.20 for the *C. jejuni *real-time PCR.

**Table 2:** Intra- and Inter-assay variabilities of *C. coli *and *C. jejuni *real-time PCR assays for the standard curves generated with purified genomic DNA of *C. coli *CIP 70.81 and *C. jejuni *NCTC 11168, ranging from 10^1 ^to 10^8 ^genome copies per PCR reaction (genome copy number) and with DNA extracted from *Campylobacter*-negative pig faecal samples spiked with different amounts of *C. coli *and *C. jejuni *ranging from 2 × 10^2 ^to 2 × 10^7 ^CFU/g of faeces including the DNA extraction procedure (CFU/g of faeces)

	**Intra-assay **^**1**^				**Inter-assay **^**2**^			
	
	*C. coli*		*C. jejuni*		*C. coli*		*C. jejuni*	
**Genome copy number**	**CV**_**c **_**(%)**	**Ct range**	**CV**_**j **_**(%)**	**Ct range**	**CV**_**c **_**(%)**	**Ct range**	**CV**_**j **_**(%)**	**Ct range**

**10**^**8**^	**2.27**	14.18-15.25	**5.63**	14.18-17.15	**4.89**	13.86-16.11	**1.94**	14.30-15.01
**10**^**7**^	**1.33**	16.63-17.71	**0.95**	17.55-18.21	**4.69**	16.33-17.88	**0.83**	17.86-18.27
**10**^**6**^	**1.99**	20.05-20.78	**1.13**	21.02-21.81	**3.42**	19.29-21.80	**1.37**	21.15-22.04
**10**^**5**^	**1.60**	23.32-24.63	**0.57**	24.15-24.69	**4.08**	23.22-25.55	**0.67**	24.01-24.48
**10**^**4**^	**0.81**	26.92-28.07	**0.35**	26.56-26.91	**1.52**	26.23-28.48	**2.65**	26.64-28.30
**10**^**3**^	**1.28**	30.44-31.28	**0.53**	30.11-30.69	**1.90**	29.70-31.37	**1.99**	28.60-30.85
**10**^**2**^	**1.22**	33-37-34.82	**0.40**	33.66-34.05	**2.46**	33.80-35.78	**1.39**	33.62-34.60
**10**^**1**^	**0.87**	37.29-38.66	**2.21**	35.65-37.77	**3.10**	37.10-38.91	**2.21**	36.11-37.43

**CFU/g of faeces**	**CV**_**c **_**(%)**	**Ct range**	**CV**_**j **_**(%)**	**Ct range**	**CV**_**c **_**(%)**	**Ct range**	**CV**_**j **_**(%)**	**Ct range**

**2 × 10**^**8**^	**3.23**	17.22-18.35	**2.28**	18-74-19.81	-	-	-	-
**2 × 10**^**7**^	**1.33**	20.60-21.15	**2.53**	20.57-22.02	**0.75**	19.54-19.88	**1.21**	21.65-22.27
**2 × 10**^**6**^	**1.89**	24.08-24.97	**0.91**	24.13-24.62	**2.37**	23.51-24.85	**0.70**	24.15-24.60
**2 × 10**^**5**^	**1.15**	27.23-28.38	**1.40**	27.02-28.45	**0.57**	26.40-26.79	**1.46**	27.04-28.69
**2 × 10**^**4**^	**2.20**	28.28-29.75	**1.98**	30.13-31.80	**2.58**	28.00-29.90	**2.10**	30.7-32.31
**2 × 10**^**3**^	**4.40**	32.20-33.77	**1.62**	34.61-35.96	**2.07**	32.00-33.22	**1.80**	34.48-36.45
**2 × 10**^**2**^	**4.38**	34.61-37.78	**1.76**	38.04-39.37	**1.64**	35.35-36.56	**1.92**	37.34-39.03

The coefficients of variation (CV) of the threshold cycles values (Ct) were evaluated for the *C. coli *real-time PCR (CV_c_) and for the *C. jejuni *real-time PCR (CV_j_). For each CV_c _and CV_j_, the range of Ct (Ct range), which corresponds to the smallest and the highest values of the Ct found among the ten, was indicated for each dilution for both intra-and inter-assay testings.

^1 ^Results of intra-assay testing: ten replicates of each sample were tested in one PCR run

^2 ^Results of inter-assay testing: one replicate of each sample was tested once in each of ten different PCR runs

### Validation of the real time PCR assays for the analysis of faecal, feed, and environmental samples spiked with C. coli and C. jejuni

Samples were checked for PCR inhibition in a separate test using a bacterial internal amplification and extraction control [34]. Inhibitors of real-time PCR were identified in 4% of the examined samples, which were consequently removed from the quantification study.

**Figure 3:**
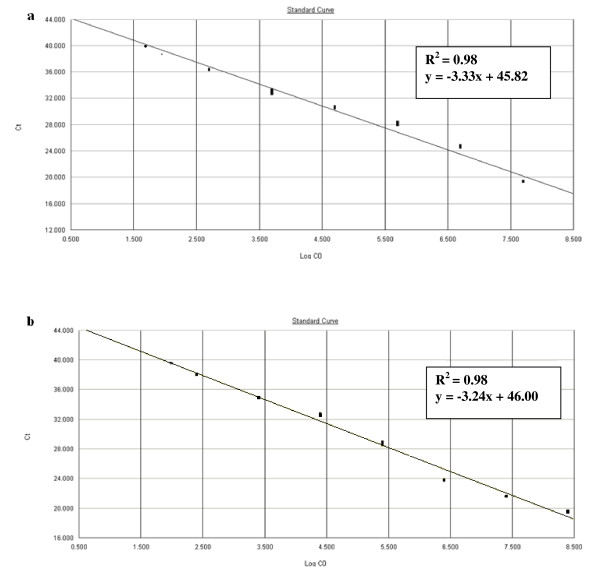
**Dynamic range and sensitivity of the *Campylobacter coli *and *Campylobacter jejuni *real-time PCR assays for faecal samples**. Standard curves of DNA extracted from the *Campylobacter*-negative faecal samples spiked with 10-fold serial dilution of (**a**) *C. coli *CIP 70.81 suspension, ranging 2.5 × 10^2 ^to 2.5 × 10^7 ^CFU/g of faeces and (**b**) *C. jejuni *NCTC 11168 suspension, ranging 2.0 × 10^2 ^to 2.0 × 10^7 ^CFU/g of faeces, each dot representing the result of duplicate amplification of each dilution. The coefficients of determination R^2 ^and the slopes of the regression curve are indicated. The standard curve is obtained by correlation of the threshold cycle values (Ct) and log_10 _input CFU/g of faeces (Log CO) from the amplification plot.

The detection limit for the quantitative real-time PCR assays in spiked faecal samples were 2.5 × 10^2 ^CFU of *C. coli*/g of faeces and 2.0 × 10^2 ^CFU of *C. jejuni*/g of faeces (Figure 3), similar to that of the bacteriological method. Although this assay was able to detect lower quantities between 5.0 × 10^1 ^and 2.0 × 10^2 ^CFU of *Campylobacter*/g of faeces, the regression curve was only linear from about 10^2 ^to 10^7 ^CFU with reaction volumes of 20 μL (Figure 3). For the feed samples, the detection limits were slightly lower (1.1 × 10^2 ^CFU of *C. coli*/g of feed and 1.3 × 10^2 ^CFU of *C. jejuni*/g of feed). For the environmental samples, they were around 10^3 ^CFU/m^2 ^for both species and both sampling sites (pen walls and floor swabs). For both species, the standard curves showed linearity from about 10^2 ^to 10^8 ^CFU and 10^3 ^to 10^7 ^CFU for feed and environmental samples respectively.

To obtain values for the intra- and inter-assay variation of each real-time PCR assay with field samples, DNA extracted from the *Campylobacter*-negative spiked faecal samples was subjected to each real-time PCR in ten duplicates, with 10 different mixes performed on different runs. The results are reported in Table 2. The CV of the Ct values for the ten different intra-assay experiments ranged from 1.15 to 4.40% for *C. coli *real-time PCR and from 0.91 to 2.53% for *C. jejuni *real-time PCR. The standard curves were *y *= -3.33*x *+ 45.82 with R^2 ^= 0.98 for *C. coli *and *y *= -3.24*x *+ 46.00 with R^2 ^= 0.98 for *C. jejuni*. The CV of the Ct values for the ten different inter-assay experiments, including the DNA extraction procedure, ranged from 0.57 to 2.58% and from 0.70 to 2.10% respectively for *C. coli *and *C. jejuni *real-time PCR assays. The mean standard curves were *y *= -3.36*x *+ 43.70 and *y *= -3.25*x *+ 46.20 respectively.

### Analysis of faecal samples of experimentally infected pigs

The numbers of positive and negative samples for experimentally infected pigs determined by either real-time PCR or bacteriological method are summarized in Table 3. There was an excellent correlation at the qualitative level with both techniques with a kappa of 0.94 and 0.89 respectively for *C. coli *and *C. jejuni *real-time PCR assays. Indeed, for *C. jejuni *experimentally infected pigs, only two culture-positive samples were negative by real-time PCR, and one culture-negative sample was positive by real-time PCR (specificity of 96.2%). In addition, for pigs experimentally infected with *C. coli*, only one culture-negative sample was positive by real-time PCR and inversely (specificity of 96.2%).

**Table 3:** Comparison of real-time PCR and microaerobic culture in faecal samples of experimentally infected pigs for the detection of (3.1) *Campylobacter coli *and (3.2) *Campylobacter jejuni*

			Microaerobic culture		
			+	-	Total
		**+**	**40**	**1**	**41**
**3.1 *Campylobacter coli *detection**	**Real-time PCR**	-	**1**	**25**	**26**
		**Total**	**41**	**26**	**67**

		**+**	**24**	**1**	**25**
**3.2 *Campylobacter jejuni *detection**	**Real-time PCR**	-	**2**	**25**	**27**
		**Total**	**26**	**26**	**52**

**3.1 **Sensitivity Se = 97.6%, Specificity Sp = 96.2%, Kappa K = 0.94

**3.2 **Sensitivity Se = 92.3%, Specificity Sp = 96.2%, Kappa K = 0.89

**Figure 4:**
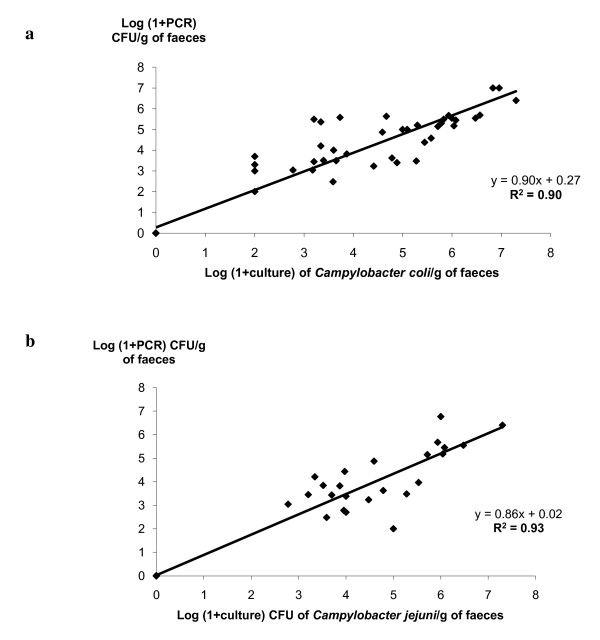
**Correlation between real-time PCR and microaerobic culture for faecal samples of *Campylobacter *experimentally infected pigs**. Scatter plot showing the differences and correlations between the real-time PCR and the microaerobic culture method for the faecal samples of pigs experimentally infected with *Campylobacter *for the detection of (a) *C. coli *and (b) *C. jejuni. *Data for *Campylobacter*-positive samples *versus Campylobacter*-negative samples by both methods fall close to the line equivalence: **a**- *Campylobacter*-positive **(*n *= 40) **and *Campylobacter*-negative **(*n *= 25) **samples respectively with a coefficient of correlation of 0.90 (R^2 ^= 0.90). **b**- *Campylobacter*-positive **(*n *= 24) **and *Campylobacter*-negative **(*n *= 25) **samples respectively with a coefficient of correlation of 0.93 (R^2 ^= 0.93).

The estimate of *Campylobacter *CFU/g of faeces by both *C. coli *and *C. jejuni *real-time PCR assays was compared to the bacteriological enumeration method (Figure 4). We observed a good correlation between real-time PCR and culture at the quantitative level for experimentally infected pig samples both for *C. coli *real-time PCR *(*R^2 ^= 0.94) and for *C. jejuni *real-time PCR *(*R^2 ^= 0.86). Among the PCR-culture positive samples for the experimentally infected pig, 72.5% of the samples had a difference in cell number of less than 1 log, 25% of less than 2 logs, and 2.5% of less than 2.5 logs for *C. coli *real-time PCR assay. For *C. jejuni *real-time PCR assay, the results obtained by real-time PCR matched equally the results obtained by culture: 67% of the samples had a difference in cell number of less than 1 log, 29% of less than 2 logs, and 4% of less than 3 logs.

### Analysis of field samples of naturally contaminated pigs

No *C. jejuni *was identified among the faecal, feed, and environmental samples from the different pig herds by conventional PCR or by our *C. jejuni *real-time PCR assay. Conversely, all the *Campylobacter *tested were identified as *C. coli *by both methods.

The specificity and the sensitivity for the *C. coli *real-time PCR assay with the different field samples are reported in Table 4.

**Table 4:** Comparison of *Campylobacter coli *real-time PCR and microaerobic culture in (4.1) faecal, (4.2) feed, and (4.3) environmental samples of naturally contaminated pigs

			Microaerobic culture		
			+	-	Total
**4.1 *Campylobacter coli *detection in faecal samples**		**+**	**125**	**1**	**126**
	**Real-time PCR**	-	**3**	**17**	**20**
		**Total**	**128**	**18**	**146**

**4.2 *Campylobacter coli *detection in feed samples**		**+**	**21**	**1**	**22**
	**Real-time PCR**	-	**2**	**26**	**28**
		**Total**	**23**	**27**	**50**

**4.3 *Campylobacter coli *detection in environmental samples**		**+**	**34**	**2**	**36**
	**Real-time PCR**	-	**3**	**47**	**50**
		**Total**	**37**	**49**	**86**

**4.1 **Sensitivity Se = 97.7%, Specificity Sp = 94.4%, Kappa K = 0.96

**4.2 **Sensitivity Se = 91.3%, Specificity Sp = 96.2%, Kappa K = 0.89

**4.3 **Sensitivity Se = 91.9%, Specificity Sp = 95.9%, Kappa K = 0.89

For the different field samples tested, the quantification results obtained by *C. coli *real-time PCR matched equally the results obtained by bacterial culture: 58% of the samples had a difference in cell number of less than 1 log, 37% of less than 2 logs, and 5% of less than 3 logs.

## Discussion

The real-time PCR asays developed in this study provide an effective, rapide, and sensitive alternative method to culture-based methods for the detection and the quantification of *C. coli* and *C. jejuni* in pure cultures and in complex samples.

To use real-time PCR for quantitative measurements and to ensure a correct quantification, information on both linear range and amplification efficiency of the real-time PCR assay must be available. With a quantitative detection limit of 10 genome copies, an amplification efficiency of 99%, and a linear range of seven to eight orders of magnitude, the *C. coli *and *C. jejuni *real-time PCR assays allowed a precise quantification of *C. coli *or *C. jejuni *DNA amounts extracted from pure culture preparations. The specificity of the assays was assessed (i) by the species-specific amplification of DNA from different field strains/isolates of *C. coli *and *C. jejuni*, and (ii) by the absence of amplification from DNA isolated from 30 pig faecal, feed, and environmental samples previously determined to be *Campylobacter*-free by culture. The real-time PCR assays were also shown to be highly specific since no PCR amplicons were detected when the method was applied to DNA from different bacterial reference strains, including different *Campylobacter *species, *Campylobacter*-related bacteria, and other bacteria. Both intra- and inter-assay coefficients of variation of the Ct values for the purified genomic DNA were satisfactorily low and in concordance with those reported for other molecular assays based on PCR amplification [35]. They confirmed the reliability and the accuracy of the technical setup over time and over the complete range of quantification.

The technique was developed to detect and quantify *C. coli *and/or *C. jejuni *directly in pig faecal, feed, and environmental samples. In order to determine the detection limits of *C. coli *and *C. jejuni *real-time PCR assays for field samples, *Campylobacter*-negative faecal samples were spiked with 10-fold dilutions of the *Campylobacter *suspensions of each reference strain (*C. jejuni *NCTC 11168 and *C. coli *CIP 70.81). Standard curves for environmental and feed samples were constructed in a similar way. The established *C. coli *and *C. jejuni *real-time PCR assays proved highly sensitive (with a quantitative detection limit of approximately 2.5 × 10^2 ^CFU/g of faeces, 1.3 × 10^2 ^CFU/g of feed, and 1.0 × 10^3 ^CFU/m^2 ^for the environmental samples) and were linear over a range of six orders of magnitude (from 2.0 × 10^2 ^to 2.0 × 10^7 ^CFU/g of faeces). Both intra- and inter-assay coefficients of variation of the Ct values for the DNA extracted from *Campylobacter*-negative faecal samples did not differ significantly. This may indicate that the main reason for variation is not due to pipetting errors in setting up the PCR assay but may be caused by contaminants from the fecal samples. Nevertheless, we did not observe systematically lower CV values of intra- and inter-assay variations with purified genomic DNA. This does not support the hypothesis that inhibitors and contaminants may interfere with uniform and consistent dilution as well as the amplification of target DNA.

Samples tested in this study constitute complex biological substrates due to the presence of (i) numerous types of bacteria, (ii) different kinds of inhibitors, and (iii) food degradation products [36,37]. Moreover, contrary to faecal and caecal chicken samples [35,38], the consistency and the composition of pig faecal samples are highly variable and heterogeneous (i) between individuals, (ii) over time according to the age of the animals, and (iii) depending on the diet components in the same way as for cattle faeces [39,40]. In this study, we sampled faeces of sows, piglets, weaners, and finishers, exhibiting considerable heterogeneity (water content, presence of mucus, and fiber content). All these variables may have an impact on the DNA extraction process and inhibitor removal, affecting the quality and the quantity of DNA obtained, thereby limiting the sensitivity of molecular studies. The modified sample preparation procedure, which included (i) a large volume of faeces (5 g fresh weight), (ii) a boiling step known to remove inhibitors of the Taq polymerase [41], and (iii) the use of a DNA extraction kit, allowed a better homogenization of the faeces and achieved partial removal of inhibitors. No difference was noticed between real-time PCR assays and culture at both qualitative and quantitative levels for faecal samples differing by the composition, the consistency, or the age of the sampled animal (data not shown). Nevertheless, in this study, the potential presence of PCR inhibitory compounds was in parallel assessed with the use of an internal bacterial control of extraction and amplification in a separate real-time PCR test [34]. Inhibitors of real-time PCR were identified only in 4% of the examined samples, which were consequently removed from the quantification study. Moreover, the DNA extraction step reproducibility, an important parameter when evaluating the DNA purification [42], was satisfactory proved with the low CV values of the inter-assay variability including the DNA extraction procedure.

Three faecal samples of experimentally infected pigs, detected as negative by PCR and direct streaking, were positive by culture after an enrichment step (one out of 41 and two out of 26 for *C. coli *and *C. jejuni *real-time PCR assays respectively) leading to a sensitivity of 97.6% and 92.3%. Although the internal control was positive, we cannot exclude the hypothesis of inhibition of *C. coli *and *C. jejuni *amplification. Indeed, it was previously reported that some PCR primers are more markedly affected than others by impurities present in DNA preparations [43,44]. Moreover, it could be false negative PCR samples, which have been below the detection limit of the two real-time PCR assays. Genetic variability among the isolates of *Campylobacter *spp., which has been demonstrated previously [45-47], can also affect the efficacy of a PCR if changes occur within one or both of the binding sites [17,46]. The enrichment step enhanced the sensitivity of the bacteriological method by lowering the detection limit. Nevertheless, even if it is helpful for poorly contaminated samples, researchers have reported several cases in which *C. jejuni *signals detected by direct PCR disappeared after enrichment. Conversely *C. coli *signals were maintained when present before enrichment, or else became detectable when undetectable before enrichment [24,48]. This suggests that the enrichment media may favour the growth of one *Campylobacter *species comparatively to the other [49].

Furthermore, for the experimentally infected pigs, only one culture-negative faecal sample was positive by real-time PCR for each target leading to a specificity of 96.2% for both *C. coli *and *C. jejuni *real-time PCR assays. These results may be due to the presence of viable but nonculturable (VBNC) forms or dead bacteria cells, since DNA-based tests detect all DNA of the extract from live as well as dead bacteria [27,29,50]. If this is the case, it is another advantage of these real-time PCR assays as *Campylobacter *cells in a VBNC state may potentially be still infectious [18,51]. The bacteriological method may also explain these results given that the sensitivity of culture may vary depending on the *Campylobacter *spp. due to differences in susceptibility to antibiotics present in selective agar [52]. Moreover, in pig faceal and environmental samples, the enrichment of *C. jejuni *could be difficult due to the presence of a high background flora and due to the more numerous *C. coli *quantity [20].

Finally, for the faecal samples of experimentally infected pigs, we observed a good correlation at the quantitative level between culture enumeration and quantitative PCR for both *C. coli *and *C. jejuni *real-time PCR assays (R^2 ^= 0.90 and R^2 ^= 0.93 respectively). Among the PCR-culture positive samples, the real-time PCR quantification seems to be accurate compared to the culture enumeration used as a gold standard. Indeed, more than 95% of the samples with a difference in cell number of less than 2 logs, of these 72.5% and 67% less than 1 log respectively for *C. coli *and *C. jejuni *real-time PCR assays. The observed discrepancy might be due to the possible presence of VBNC forms, dead cells and antagonistic bacterial species. Another possibility could be the impact of dilution factors used for quantitative culture or an insufficient homogenization of the samples. This method provides a mean to identify and quantify at the species level *C. coli *and *C. jejuni *directly from faecal, feed, and environmental samples without requiring an enrichment step. For the different field samples tested, the qualitative data (specificity and sensitivity) as well as the quantification results obtained by *C. coli *real-time PCR matched equally the results obtained by bacterial culture. In this study, no *C. jejuni *was identified among the faecal, feed, and environmental samples from the different pig herds by conventional PCR or by our *C. jejuni *real-time PCR assay. Conversely, all the *Campylobacter *tested were identified as *C. coli *by both methods. In France, pigs were found to be almost always contaminated by *C. coli*, these first results confirmed this predominance. Nevertheless, given that we can find both species in pigs [10,12-14], these real-time PCR assays allow a direct and rapid investigation of the carriage and the excretion of *C. coli *and *C. jejuni *in conventional pigs.

## Conclusion

The real-time PCR assays for *C. coli *and *C. jejuni *described in this study have several advantages over culture-based techniques. These include allowing a large increase in throughput, enabling simultaneous processing of several samples (the real-time PCR can be run in a 96-well format and many steps in the assay can be automated), and reducing the total time required for analysis. The identification at the species level and the quantification on the entire DNA extracted from faecal, feed, and environmental samples is a new tool to enhance our understanding of the epidemiology of *Campylobacter*. In terms of risk assessment, this ability to differentiate and quantify these two species permits a more precise description of the carriage and excretion of *C. coli *and *C. jejuni *by livestock animals.

## Methods

### Bacterial strains and culture conditions

Different *Campylobacter *spp., *Helicobacter, Wolinella*, and *Arcobacter *reference strains were used to test the specificity of primers and probes for real-time PCR identification and differentiation of *C. coli *and *C. jejuni *(Table 1). In addition, we have tested 50 *C. jejuni *and 75 *C. coli *isolates (from human, poultry, and pig origin) as well as other enteric bacteria (clinical isolates and reference strains) selected from our in-house collection, the collection of the French Agency for Food, Environmental and occupational Health and Safety (Anses, Ploufragan), and the collection of the French National Reference Center for *Campylobacter *and *Helicobacter *(CNR-CH, Bordeaux). Strains were stored at -80°C in brain heart infusion broth (Difco, Detroit, Michigan) containing 20% (v/v) glycerol. Moreover, for the real-time PCR reactions, we used the two reference strains *C. jejuni *NCTC 11168 and *C. coli *CIP 70.81 as positive controls as well as *Listeria monocytogenes *ATCC 19115 and *Escherichia coli *CIP V517 as negative controls. *Campylobacter *strains were grown at 25, 37 or 41.5°C for 48 h in a microaerobic atmosphere (5% O_2_, 10% CO_2_, 85% N_2_) on Karmali agar plates (Oxoid, Dardilly, France). *Arcobacter, Helicobacter*, and *Wolinella *were grown at 37°C for 48 h on Columbia Blood agar plates (Oxoid, Dardilly, France) with 5% of defibrinated sheep blood (AES Chemunex, Combourg, France) and *Enterobacter aerogenes *on Purple Lactose agar plates (BCP, AES Chemunex, Combourg, France) for 24 h. All the other bacteria listed in Table 1 were grown under appropriate culture conditions at 30 or 37°C for 24 h on Tryptone soja agar yeast extract plates (TSAYE Oxoid, Dardilly, France).

### DNA extraction from bacterial cultures

Genomic DNA from each bacterial culture was extracted using the Nucleospin^® ^Tissue mini-kit (Macherey Nagel, Hoerdt, France) and according to the manufacturer's instructions. The concentration of isolated double stranded DNA was determined by measuring the optical density at 260 nm with the Spectronic^® ^Genesys™ 5 (Spectronic Instruments Inc., New York, USA). The purity was assessed by the examination of 260/280 nm optical density ratios [53]. All DNA samples classified as pure (*i.e. *having a 260/280 nm optical density ratio between 1.8 and 2.0) were adjusted to 20 ng μL^-1 ^in TE buffer (10 mmol Tris-HCl, 1 mmol EDTA, pH 7.6) and stored at -20°C until required for analysis.

### Construction of the standard curves with purified genomic DNA

Total genomic DNA of *C. jejuni *NCTC 11168 and *C. coli *CIP 70.81 cultures were extracted as described above. The genome copy numbers of *C. jejuni *and *C. coli *in 100 ng of DNA (for one PCR reaction) was calculated on the basis of the genome size (1 640 Kbp for *C. jejuni*, 1 860 Kbp for *C. coli*) [54-56] and was equal to 5.2 × 10^7 ^and 4.6 × 10^7 ^copies respectively. After DNA quantitation by spectrofotometrical analysis with the Spectronic^® ^Genesys™ 5, 10-fold dilutions of each extract were produced in TE buffer, representing 10^1 ^to 10^8 ^genome copies of *C. jejuni *per 5 μL of template (PCR reaction) and 0.3 × 10^1 ^to 3.0 × 10^8 ^genome copies of *C. coli *per 5 μL of template (PCR reaction). Moreover, a standard curve with roughly equal genome copies of *C. jejuni *and *C. coli *together was produced for each PCR assay. Serial DNA dilutions were aliquoted: some were stocked at 4°C to be use directly, others were stored frozen at -20°C and thawed once for use.

### Sample collection

#### *Campylobacter-*negative samples

Fifteen *Campylobacter-*negative faecal samples were obtained from specific pathogen-free (SPF) sows and piglets from the high-security barn at the Anses centre (Ploufragan, France). Moreover, five *Campylobacter-*negative feed samples and 10 *Campylobacter-*negative environmental samples were collected from the same high-security barns. These samples were used to test the specificity and/or the analytical sensitivity of the real-time PCR assays. For the environmental samples, each pen of pigs was sampled on the bottom of the wall and pen partitions using swabs (sterile square pieces of cotton cloth (32 . 32 cm) moistened with isotonic saline solution) (Sodibox, La Forêt-Fouesnant, France). The swabs were placed in a sterile bag before to be analyzed.

#### Additional faecal, feed, and environmental samples

Faecal samples were obtained from both pigs experimentally inoculated with 5 × 10^7 ^CFU of *Campylobacter *(*n *= 119, respectively 67 *C. coli *and 52 *C. jejuni *faecal samples) [57] and naturally contaminated pigs in five conventional herds (*n *= 146). Given that pig faeces can be highly heterogeneous, rectal faecal samples were collected individually from naturally contaminated (i) sows, (ii) pigs aged three to 16 weeks, and (iii) piglets aged one to three weeks. Additionally, in the five conventional herds, 86 environmental swabs of pig pens (either empty or with animals) and 50 feed samples were collected. The swabbed surface area was measured each time.

#### Sample processing and experimental conditions

All samples were examined within four hours after sampling for *Campylobacter *spp. quantification by conventional culture and for species-identification by the PCR described by Denis *et al. *(1999) [24] as well as for species-specific quantification by real-time PCR assays. All animals of this study were housed and treated in accordance with the regulations of the local veterinary office (Direction des Services Vétérinaires des Côtes d'Armor, France). The animal experimention was carried out following the international recognized guidelines. All the animals were reared in isolation rooms with controlled air flow [57].

### DNA preparation for real-time PCR-based quantification

DNA isolation from the faecal, feed, and environmental samples was performed using a modified extraction protocol of the Nucleospin^® ^Tissue mini-kit (Macherey Nagel, Hoerdt, France) with a preliminary step of boiling to remove inhibitors of the Taq polymerase [41]. Five grams of sample (faeces or feed) were diluted in 5 mL of sterile water (for smaller amounts, an equivalent quantity of sterile water (w/w) was added). The environmental swabs, placed into sterile bags, were stomached for 2 min with 10 mL of sterile water. The sample solutions of faeces, feed, and swabs were boiled for 10 min, chilled on ice, and centrifuged (8000 *g*, 5 min). For each sample, 250 μL of supernatant was extracted using the Nucleospin^® ^Tissue mini-kit according to the manufacturer's instructions. Finally, DNA preparations, eluted in 100 μL of elution buffer purchased in the kit, were stored at +4°C prior to use.

### Control of PCR inhibition

To test the presence of PCR inhibitors in the DNA isolated from the samples, a fixed amount of the bacterium *Yersinia ruckeri *was added to each sample before the DNA extraction. This internal bacterial amplification and extraction control was quantified in a separate well using a real-time PCR test described in a previous work [34]. Samples with PCR inhibition were then removed for the rest of the study.

### Enumeration of Campylobacter spp. and species identification

Ten grams of fresh faeces, ten grams of feed, and the environmental swabs were vortexed in 90 mL of Preston broth (Oxoid, Dardilly, France) with a Preston antibiotic supplement (Oxoid, Dardilly, France) (for rectal swabs, 9 mL of Preston broth was added to one gram of faeces). For *Campylobacter *numeration, 100 μL of a ten-fold dilution serie (10^-1 ^to 10^-5^) were plated both on Karmali agar (Oxoid, Dardilly, France) and on Butzler agar (Oxoid, Dardilly, France) and incubated for 24 to 72 h at 41.5°C in microaerobic conditions. To promote the detection of *Campylobacter *in samples harbouring less than 100 Colony Forming Unit of *Campylobacter *per gram of faeces (CFU/g of faeces), a second plating was carried out 24 h after broth enrichment and incubated 48 h. Thermophilic *Campylobacter *were cultured at 41.5°C in microaerobic conditions. For direct streaking and selective enrichment, the *Campylobacter *suspect colonies on Karmali or Butzler plates were confirmed by microscopy (cell morphology) and conventional PCR [24]). The number of CFU/g of faeces or feed as well as the number of CFU/m^2 ^for the environmental samples were thus calculated. Finally, material from *Campylobacter *suspect colonies was suspended in TE buffer and subdued to DNA extraction and the species-specific PCR described by Denis *et al. *(1999) [24] for differentiation between *C. coli *and *C. jejuni*.

### Real-time PCR primers and probes

To detect *C. jejuni *and *C. coli*, we have used sequences described by Lagier *et al. *(2004) [33], which are based (i) on the single-copy *hipO *gene (benzoylglycine amidohydrolase) responsible for the hippurate activity exclusively found within the *C. jejuni *genome, and (ii) on the single-copy *glyA *gene (serine hydroxymethyltransferase) in an unique nucleotide region within the *C. coli glyA *open reading frame identified as specific for *C. coli *[58] (Table 5).

**Table 5:** PCR primers and probes used in the species-specific real-time PCR assays

**Primer or Probe**^**a**^	Nucleotide sequence 5'-3'	Location within target	Origin	**Target Gene detected**^**b**^
*glyA*-F forward	F: AAACCAAAGCTTATCGTGTGC	297-320	This study	
*glyA*-R reverse	R: AGTGCAGCAATGTGTGCAATG	422-359	Lagier *et al. *(2004)	*Campylobacter coli glyA *gene (125 bp)
*glyA*-P MGB Probe	P: **FAM**-CAACTTCATCCGCAAT	346-330	This study	

*hipO*-F forward	F: CTTGCGGTCATGCTGGACATAC	340-360	This study	
*hipO*-R reverse	R: AGCACCACCCAAACCCTCTTCA	464-444	This study	*Campylobacter jejuni hipO *gene (124 bp)
*hipO*-P MGB Probe	P: **VIC**-ATTGCTTGCTGCAAAGT	424-409	This study	

bp, length in base pairs of the species specific PCR products

^a^Primers and probes were designed by using the program Primer Express version 2.0 (Applied Biosystems, Foster city, CA, USA). The TaqMan^® ^MGB probes were dual-labelled with either fluorescent reporter dyes **FAM **(6-carboxyfluorescein, *C. coli *specific probe) or **VIC **(*C. jejuni *specific probe) on the 5'end, and quenched by a non fluorescent quencher associated with a minor groove binder at the 3'end (Applied Biosystems).

^b^The nucleotide sequences were retrieved from the GenBank™ sequence database http://www.ncbi.nlm.nih.gov/Genbank/index.html under accession numbers: [GenBank: Z36940] for *C. jejuni hipO *gene and [GenBank: AF136494] for *C. coli glyA *gene.

To optimize the real-time PCR reaction and to improve the specificity and the mismatch discrimination, shorter Minor Groove Binder (MgB) probes have been designed [59-62]. At the 5'end, the *C. coli *probe was linked to the fluorophore FAM and the *C. jejuni *probe to the fluorophore VIC. The *C. jejuni *and *C. coli *species-specific primers and TaqMan^® ^MgB probe sets were thus designed with Primer Express software version 2.0 (Applied Biosystems, Foster city, CA, USA) according to the recommendations of the manufacturer (Table 5). This software was used to choose the best combinations of each primers-probe set values. Finally, the selected primers and probes were checked for homology to non-target sequences by a search with the BLAST program of the National Center for Biotechnology Information (NCBI). Primers and MgB probes were synthesized by Applied Biosystems and stored at -20°C prior to use.

### Real-time PCR amplification

Reactions were done in 20 μL PCR mixtures containing 10 μL of 1X Taqman Universal PCR Mastermix (AmpliTaq Gold™ DNA polymerase, dNTPs, Passive reference (ROX), and optimised buffer components including 5 mM MgCl_2_), 400 nM of each primer (*gly*A-R and *gly*A-F for *C. coli *real-time PCR assay, *hip*O-R and *hip*O-F for *C. jejuni *real-time PCR assay), 200 nM of the probe (*gly*A-P and *hip*O-P respectively), and 5 μL of template DNA. The thermal cycle protocol used was the following: activation of the Taq DNA polymerase at 95°C for 10 min, then 45 or 48 cycles of 15 s at 95°C and 60 s at 60°C. Thermal cycling, fluorescent data collection, and data analysis were carried out with the ABI PRISM^® ^7300 Sequence Detection System (Applied Biosystems) according to the manufacturer's instructions. Fluorescence of FAM and VIC was measured at their respective wavelengths during the annealing/elongation step of each cycle. After real-time data acquisition, the baseline cycles for the FAM and VIC signals were set from cycle three to three cycles below the cycle at which the first signal appeared and the threshold value at the point at which the fluorescence exceeded 10 times the standard deviation of the mean baseline emission. The threshold cycle (Ct) is the first PCR cycle at which a statistically significant increase in fluorescent signal is detected. All reactions were carried out alongside a non template control containing all reagents except DNA, positive controls containing DNA from reference strains (*C. jejuni *NCTC 11168 and/or *C. coli *CIP 70.81), and negative controls containing DNA from *Listeria monocytogenes *ATCC 19115 and from *Escherichia coli *CIP V517. All the DNA extractions were done as described before. Each control was run in triplicate and each sample in duplicate.

### Evaluation of performance of the real-time PCR assays

#### Specificity and sensitivity

The specificity of each real-time PCR assay was first assessed with purified genomic DNA preparations (about 10^6 ^genome copies per PCR reaction) of different bacterial strains (Table 1) and then with DNA extracted from 30 *Campylobacter-*negative faecal, feed, and environmental samples as defined above. This screening strategy, described previously by Lagier *et al. *(2004) [33], ensure the specificity of the primers and probes for *C. jejuni *and *C. coli *only in field samples. Double-stranded DNA extracted from the 30 *Campylobacter*-negative faecal, feed, and environmental samples was examined in duplicate with each real-time PCR assay.

The sensitivity for each PCR assay was determined using the standard curves prepared with purified genomic DNA of cultures of *C. jejuni *NCTC 11168 and *C. coli *CIP 70.81, ranging from 10^1 ^to 10^8 ^genome copies per 5 μL of template (PCR reaction). In order to mimic realistic conditions and to determine the detection limits of *C. coli *and *C. jejuni *real-time PCR assays for field samples, different standard curves were prepared to quantify *C. coli *or *C. jejuni *in faecal, feed, and environmental samples. *Campylobacter*-negative faecal samples were spiked with 10-fold dilutions series of viable suspensions of each reference strain (*C. jejuni *NCTC 11168 and *C. coli *CIP 70.81), ranging from 10^1 ^to 10^8 ^Colony Forming Units per gram of faeces (CFU/g). Standard curves for environmental and feed samples were constructed in a similar way. DNA was extracted from each of the spiked samples and tested in real-time PCR, where the standard curves were created automatically by the ABI PRISM^® ^7300 Sequence Detection System Software by plotting the Ct values against each standard dilution of known concentration.

#### Intra- and inter- assay variabilities

The assay variability was established by repeatedly testing samples containing several concentrations of *C. coli *and *C. jejuni *spanning the whole range covered by each real-time PCR in different assays (10 consecutive runs) and within an assay (10 duplicates in the same assay), in order to calculate the inter- and intra-assay coefficients of variation (CV) for the Ct values experimentally determined, as previously described [63].

To assess the intra-assay variation, each dilution of purified genomic DNA of cultures from *C. jejuni *NCTC 11168 and *C. coli *CIP 70.81 from approximately 10^1 ^to 10^8 ^CFU were measured 10 times each within one PCR run. The inter-assay variation was evaluated with the same different dilutions of purified genomic DNA in 10 independent PCR experiments on different days (10 different runs). For each PCR run, each dilution point was tested in duplicate and the mean standard curve was used for quantity estimation.

To assess the method with field samples, the values for the intra- and inter-assay variations of the real-time PCR assays were obtained with the DNA extracted from the *Campylobacter*-negative spiked samples. To assess the intra-assay variation, DNA extracted from the *Campylobacter*-negative faecal samples spiked with 10-fold dilutions of the *Campylobacter *suspensions, ranging from 2.5 × 10^7 ^to 2.5 × 10^2 ^CFU of *C. coli*/g of faeces and from 2.0 × 10^7 ^to 2.0 × 10^2 ^CFU of *C. jejuni*/g of faeces, were measured 10 times each within one real-time PCR run. The inter-assay variation was evaluated with different dilutions of DNA extracted each time with a specific extraction from the *Campylobacter*-negative spiked faecal samples in 10 independent real-time PCR experiments on different days. For each real-time PCR run (*C. coli *and *C. jejuni *real-time PCR assays), each dilution point was tested in duplicate and the mean standard curves were used for quantity estimation. The CV of the Ct values were calculated for the ten different inter-assay experiments. They illustrate the variability of the Ct values obtained between experiments including the specific DNA extraction procedure and the amplification step.

#### Use of the standard curves

The standard curves were thus used (i) to evaluate the sensitivity of the real-time PCR assays, (ii) to assess the intra- and inter-assay variabilities, and (iii) to allow a reliable quantification of *C. jejuni *and *C. coli *in pure cultures or in the field samples.

### Statistical analysis

PCR amplification efficiency (E) was estimated using the slope of the standard curve and the formula E = 10^(-1/slope)^-1. A reaction with 100% efficiency will generate a slope of -3.32. Data analysis was performed using the SDS software (Applied Biosystems).

The 119 field samples from the experimental infection were evaluated in parallel with the real-time PCR assays and the bacterial culture described in this study. All data analyses were performed with Microsoft excel and SAS Systems version 8 (SAS, Cary, N.C.). Specificity and sensitivity were assessed using the bacterial culture as a gold standard. The sensitivity was calculated as *a*/(*a*+*c*), where *a *is the number of samples found positive by both real-time PCR and bacterial culture (direct inoculation or after selective enrichment) and *c *is the number of samples positive by bacterial culture but negative by real-time PCR. The specificity was calculated as *d*/(*b*+*d*), where *d *is the number of samples negative by both methods and *b *is the number of samples positive by real-time PCR but negative by bacterial culture. Kappa-statistic was used to measure the agreement between the microaerobic cultivation and each species-specific real-time PCR assay [64].

## Authors' contributions

MLM participated in the design of the study, the collection of study samples, and in the microbiological analysis; carried out the molecular genetic studies, designed the specific oligonucleotides, participated in the sequence alignment, and drafted the manuscript. MD was responsible for the experimental infection, participated in the collection and microbiological analysis of study samples, and helped to draft the manuscript. FB performed the statistical analysis, and helped to draft the manuscript. HS helped to draft the manuscript. CB participated in the study conception and coordination, provided guidance during all parts of the work, and helped to draft the manuscript. All authors read and approved the final manuscript.
